# Contact Lens Wear Induces Alterations of Lactoferrin Functionality in Human Tears

**DOI:** 10.3390/pharmaceutics14102188

**Published:** 2022-10-14

**Authors:** Erika Ponzini, Silvia Tavazzi, Giacomo Musile, Franco Tagliaro, Rita Grandori, Carlo Santambrogio

**Affiliations:** 1 Materials Science Department, University of Milano Bicocca, 20125 Milan, Italy; 2 COMiB Research Center, University of Milano Bicocca, 20125 Milan, Italy; 3 Unit of Forensic Medicine, Department of Diagnostics and Public Health, University of Verona, 37134 Verona, Italy; 4 Institute of Translational Medicine and Biotechnology, Sechenov First Moscow State Medical University, 119146 Moscow, Russia; 5 Biotechnology and Biosciences Department, University of Milano Bicocca, 20126 Milan, Italy

**Keywords:** unprocessed tears analysis, soft contact lenses, lactoferrin unfolding, terbium fluorescence, protein-metal ion binding analysis

## Abstract

The tear film is a complex matrix composed of several molecular classes, from small metal ions to macromolecules. Contact lens (CL) wear can affect the protein homeostasis of the tear film, by accumulating deposits on the CL surface and/or altering their structural and functional properties. This work investigates the effect of CL wear on lactoferrin (Lf), one of the most abundant tear proteins, known as an unspecific biomarker of inflammation. Tears from eight volunteers were collected and analyzed after alternated periods of CL wear and without CL. The experimental approach is to probe Lf into unprocessed human tears by the peculiar fluorescence emission originating from complex formation of Lf with terbium (Tb^3+^) at the iron-binding sites. The experimental data indicate that CL wear does not significantly affect the total amount of Lf. On the other hand, Lf affinity for Tb^3+^ is reduced upon CL wear, suggesting relevant changes in Lf structure and possible alterations of protein functionality. Future studies based on this approach will help define CL features (material, lens-care solution, wearing time, etc.) with minimal effects on tear protein activity, in order to obtain more biocompatible and comfortable devices.

## 1. Introduction

The basal tear film contains a wide variety of molecules, including proteins, peptides, lipids, metabolites, and electrolytes. The tear proteome contains more than one thousand proteins, mainly involved in the eye defense from external pathogens [1,2,3]. Lactoferrin (Lf)—also known as lactotransferrin—is a 80-kDa glycoprotein, among the most highly represented proteins (~25% of the total tear protein content) [2,3,4,5] together with lysozyme, immunoglobulins, lipocalin, and albumin [2,3]. Lf acts as an antimicrobial and anti-inflammatory agent, as well as a free-radical scavenger [4,5], and has also been recently identified as an effective inhibitor against coronavirus infection [6,7,8,9]. Due to its wide range of biological activities, Lf concentration in tears is finely modulated under different physio-pathological states and has been proposed as a possible diagnostic biomarker of dry eye disease [4].

The wearing of contact lenses (CLs) can lead to possible alterations of the tear film. In particular, proteins can penetrate into the CLs matrix in the order of a few micrograms, depending on wear time, lens-care solution, and CL material [10,11]. Generally, silicone hydrogel CLs present low protein and high lipid deposition, whereas conventional anionic hydrogel materials—like Etafilcon A CLs—show the opposite propensity [2,3,12]. Most of these studies make use of artificial tears solutions (ATSs), a simplified version of real tears containing several salts, lipids, and proteins. ATSs have the advantage to be simple, highly controlled, and reproducible samples for in vitro analyses, but represent only a rough model of the CLs–tears interaction [13,14].

Upon CLs adsorption, tear proteins may encounter structural changes [15]. The extent of these changes depends on the chemical properties of the CL material and its surface morphology [16,17,18]. These structural changes can, in turn, affect the biological activity of the protein and lead to physiological dysfunctions. Enzymatic activity assays, such as micrococcal turbidity assay, have been employed in the case of lysozyme to assess the functionality of the protein adsorbed on CLs, using ATSs [19] or real tears [20]. The results show that CL materials and lens care systems have critical roles for the retention of the conformational properties and functionality. Notably, one report highlighted an association between the amount of denatured lysozyme and subjective discomfort after CL wear [21], whereas no strong correlation has been found between the amount of protein deposited on CLs and adverse effects [22].

The investigation of CL-induced protein dysfunction, however, needs to be extended to other important tear species that are involved in ocular or systemic pathologies and are, thus, potential biomarkers. In addition, the analysis should not be limited to protein molecules deposited on the CL, since also unfolded molecules remaining in the tear solution upon CL interaction could lead to severe dysfunctional processes [23]. Finally, the complexity of the tear film [24] is not adequately modeled by ATSs, suggesting that experimental approaches with real, unprocessed human tears are necessary.

In this work, the effect of CL wear on Lf was investigated by analyzing the tears of several volunteers. Recent studies have developed biosensors for tear Lf quantification based on fluorescence detection of Lf-terbium (Tb^3+^) complexes [25]. Indeed, unsaturated Lf in tears can bind Tb^3+^ in its iron-binding sites [26], forming a fluorescent complex that can be used for specific protein detection [25]. The fluorescence properties of the Lf-Tb^3+^ complex is exploited here for direct in-bulk detection and functional characterization of Lf in unprocessed human tears. In particular, the method is employed to assess Lf amount and functional properties upon conventional hydrogel CL (Etafilcon A) wearing.

## 2. Materials and Methods

### 2.1. Materials

All chemical reagents were analytical grade and purchased from Sigma-Aldrich (St. Louis, MO, USA). Lf was purchased from MyBiosource (San Diego, CA, USA). Ultrapure water, filtered on a 0.22-µm pore membrane, was used to prepare all the solutions.

### 2.2. Fluorescence Detection

The Tb^3+^ fluorescence setup, already optimized in previous works for transferrin (Tf) [27,28,29]—the Lf serum homologous—was reproduced here for commercial Lf. Standard solutions of Lf were prepared by solubilization of commercial Lf powders (Apo-Lf and Fe^3+^-Lf) in 20 mM ammonium bicarbonate, pH 8, and 100 mM ammonium acetate. All samples were incubated at 20 °C for 10 min immediately prior to fluorescence analysis, in the absence or presence of TbCl_3_. Fluorescence measurements were carried out on a Cary Eclipse instrument (Varian, Palo Alto, CA, USA) at 20 °C, in a quartz cell of 1 cm pathlength. The excitation wavelength was set at 295 nm (bandwidth 5 nm) and emission was monitored in the range 450—575 nm, with the photomultiplier set at 800 V. The final fluorescence intensity values (expressed in arbitrary units, a.u.) were corrected by the blank and, for titration experiments, the corresponding dilution factor.

### 2.3. Tear Samples Collection

Regarding the analyses on human tears, eight healthy participants from 20 to 27 years of age were recruited for a preliminary investigation on a small pool of volunteers. Inclusion criteria were the absence of any ocular or systemic pathology, normal weight, and the absence of any medical therapy. The study was conducted following the Declaration of Helsinki ethical principles and was approved by the competent institutional human experimentation committee (approbation N° 0055071/19, 11 July 2019). The participants were habitual CL wearers. At the beginning of the study, participants were asked not to wear CLs for seven days (week n°1) and not to use lacrimal substitutes or other similar products. The participants were then instructed to wear daily disposable Etafilcon A hydrogel CLs (1-DAY Acuvue MOIST, Johnson & Johnson) for a period of one week (week n°2). Finally, the volunteers did not wear any kind of CLs for the subsequent seven days (week n°3). Collection of tears was carried out at the end of weeks n°2 and n°3. For each volunteer, a sample volume of 5 µL was collected from each eye using the capillary method [3], for a total specimen volume of 10 µL per participant. Care was taken in order to minimize eye irritation during the procedure by avoiding ocular surface contact with the capillary. Two distinct pools with a final volume of 40 µL each were obtained by mixing tear samples from four participants per pool. After collection, the samples were immediately stored at −80 °C until analysis [3,30]. Prior to analysis, the tear samples were incubated at 20 °C for a period of 10 min and 40 µL tear samples were split into two 20 µL aliquots, in order to obtain technical duplicates. Then, 20 µL of tear samples were diluted to a final volume of 150 µL in an aqueous solution of 20 mM ammonium bicarbonate, pH 8, and 100 mM ammonium acetate.

## 3. Results

### 3.1. Tb^3+^-Lf Complex

**Figure 1:**
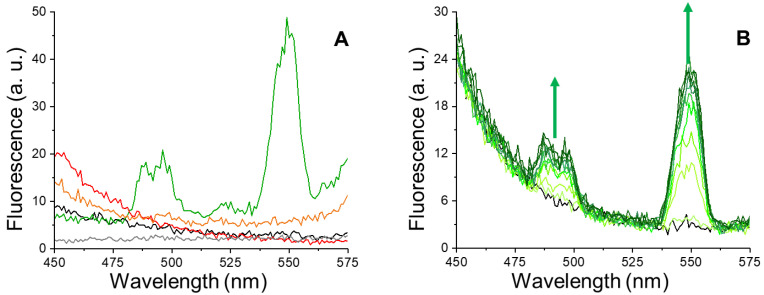
Lf-Tb^3+^ fluorescence spectroscopy. (**A**) Fluorescence emission spectra of commercial Apo-Lf (black line) and Fe^3+^-Lf (red line) in the absence of TbCl_3_. Emission spectra of Apo-Lf (green line) and Fe^3+^-Lf (orange line) in the presence of 150 µM TbCl_3_. The spectrum of a 150 µM TbCl_3_ solution in the absence of proteins is also reported (gray line). (**B**) Representative fluorescence emission spectra of pooled tear samples in the presence of TbCl_3_ (0–150 µM). Green arrows indicate the direction of spectral changes at increasing concentrations of terbium.

Intrinsic fluorescence of Apo-Lf and Fe^3+^-Lf solutions is shown in Figure 1A (black and red lines, respectively). As expected, the spectra in the absence of Tb^3+^ show no emission peaks in the selected detection range. On the contrary, the addition of TbCl_3_ to the Apo-Lf solution results in the appearance of two new peaks centered at about 490 nm and 550 nm (Figure 1A, green line). These peaks are characteristic of Tb^3+^ fluorescence emission and are in line with previous observations on Tf-Fe^3+^ complexes [29]. Accordingly, these signals are not present when TbCl_3_ solutions are analyzed in the absence of protein (Figure 1A, gray line). Thus, these peaks are diagnostic of Lf-Tb^3+^ complexes. These peaks are not observed for Fe^3+^-Lf solutions in the presence of TbCl_3_ (Figure 1A, orange line), suggesting that Tb^3+^ binds to the same Lf site as iron, but with lower affinity. This is in agreement with previous reports [26] and in analogy to what was observed for Tf [29].

### 3.2. Lf Titrations by Tb^3+^ in Row Human Tears

After these analyses on commercial Lf, human tear specimens were incubated with TbCl_3_ (0–150 µM) to test whether this assay can be applied to Apo-Lf detection in unprocessed tear samples. The resulting fluorescence spectra show the diagnostic emission peaks of Lf-Tb^3+^ complexes (Figure 1B, green lines), which are not present in the absence of TbCl_3_ (Figure 1B, black line). These results are indicative of complex formation and also confirm that a fraction of tear Lf is not loaded by iron, as suggested by previous studies [31].

**Figure 2:**
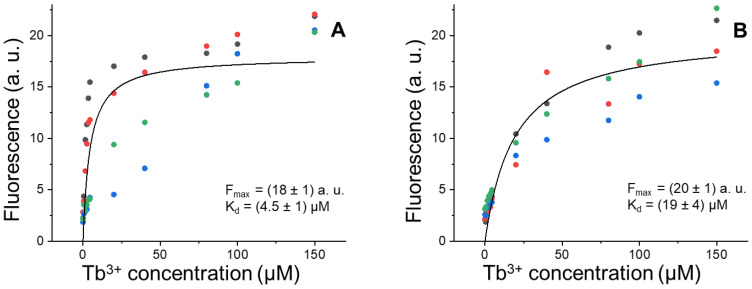
Lf-Tb^3+^ binding properties. Tb^3+^ titration of pooled tears samples after one week without CLs (**A**) and one week of CLs wearing (**B**). Fluorescence emission at 550 nm is reported. Data points of four experimental sets for each condition are indicated with distinct colors. Global fitting of the experimental sets with Equation (1) is shown as a black curve and the resulting fitting parameters are reported in each panel.

Titration experiments on tear samples obtained from the volunteers after week n°2 (daily CLs wearing) and week n°3 (no CLs wearing) were carried out in order to assess the binding parameters of the Lf-Tb^3+^ complex. Data of fluorescence emission vs. Tb^3+^ concentration were analyzed by a global fitting procedure, employing the binding curve described by the equation:

(1) $$y=\frac{F_{\max}\;\cdot \;x}{\left(K_{d}+x\right)\;}$$

where F_max_ is the fluorescence intensity under saturation conditions (excess of Tb^3+^ with respect to Apo-Lf), and K_d_ is the dissociation constant of the complex (Figure 2).

Statistical significance of the changes in F_max_ and K_d_ between the two conditions was evaluated by non-parametric bootstrapping. F_max_ is the plateau value of the binding curve and is proportional to the amount of Apo-Lf present in the sample. The results of Figure 2 show that CL wear for one week does not appreciably change Lf levels (F_max_ = 20 ± 1 with CLs and 18 ± 1 without CLs). This finding is consistent with previous reports, which highlighted a significant change of protein amount upon CL wearing only for prolactin-induced protein [32]. On the other hand, CLs have a remarkable effect on the apparent affinity of Lf for Tb^3+^ (K_d_ = 19 ± 4 µM with CLs and 4.5 ± 1 µM without CLs) (Figure 2). This four-fold increase in K_d_ upon CLs wearing likely reflects Lf structural changes that compromise the metal-ion binding sites of the protein, with possible dysfunctional implications in Lf activity as an antimicrobial and anti-inflammatory agent, as well as a free-radical scavenger.

## 4. Discussion

A comprehensive understanding of tear protein adsorption on CLs, including the possible protein unfolding caused by CL wearing, is mandatory for the production of novel CL materials that are fully biocompatible and meet the needs of the end user, such as duration of wear, comfort, etc. [33]. In this work, a fast analytical method based on Tb^3+^-induced fluorescence was developed to selectively probe Apo-Lf levels and functional state directly in the tear specimen, with minimal sample manipulation and without fractionation. To the best of the authors’ knowledge, this is the first study showing functional alteration by CL wear of a protein distinct to lysozyme. Further studies based on a higher number of participants may expand the understanding of Lf implication in the response to CL wear time, CL care solutions, and CL materials. Protein inactivation could be due to alterations of the protein conformational states and trigger an inflammatory response [34,35]. Indeed, high levels of protein denaturation have been found to be correlated with adverse effects, such as papillary conjunctivitis [36] and CL discomfort [21]. Improving biocompatibility is one of the main goals of CL manufacturers and controlling protein alterations represents a major issue. Indeed, high CL biocompatibility implies that protein–CL interaction does not involve alterations of the protein structural properties necessary for their biological activity. In this view, an optimized CL material should bind proteins mainly reversibly and maintain their native functional state [37].

## Data Availability

The data presented in this study are available on request from the corresponding author.
